# Clinical features and characteristics of *Clostridium difficile* PCR-ribotype 176 infection: results from a 1-year university hospital internal ward study

**DOI:** 10.1186/s12941-015-0114-0

**Published:** 2015-12-23

**Authors:** Jiri Drabek, Otakar Nyc, Marcela Krutova, Jan Stovicek, Jana Matejkova, Radan Keil

**Affiliations:** Department of Internal Medicine, 2nd Faculty of Medicine, Charles University in Prague and Motol University Hospital, 150 06 V Uvalu 84, Praha 5, Prague, Czech Republic; Department of Medical Microbiology, 2nd Faculty of Medicine, Charles University in Prague and Motol University Hospital, Prague, Czech Republic; DNA Laboratory, Department of Paediatric Neurology, 2nd Faculty of Medicine, Charles University in Prague and Motol University Hospital, Prague, Czech Republic

**Keywords:** *Clostridium difficile*, PCR-ribotype 176, Horn’s index, ATLAS score, Ribotyping, MLVA

## Abstract

**Background:**

*Clostridium difficile* infection (CDI) is a major cause of antibiotic-associated diarrhoea. Given an increasing CDI incidence and global spread of epidemic ribotypes, a 1-year study was performed to analyse the molecular characteristics of *C. difficile* isolates and associated clinical outcomes from patients diagnosed with CDI in the Internal Medicine department at University Hospital Motol, Prague from February 2013 to February 2014.

**Results:**

A total of 85 unformed stool samples were analysed and CDI was laboratory confirmed in 30 patients (6.8 CDI cases per 10,000 patient bed days and 50.6 CDI cases per 10,000 admissions). The CDI recurrence rate within 3 months of treatment discontinuation was 13.3% (4/30). Mortality within 3 months after first CDI episode was 26.7% (8/30), with CDI the cause of death in two cases. 51.9% of *C. difficile* isolates belonged to PCR-ribotype 176. MLVA of ribotype 176 isolates revealed two clonal complexes formed by 10/14 isolates. ATLAS scores and Horn’s index were higher in patients with ribotype 176 infections than with non-ribotype 176 infections.

**Conclusion:**

This study highlights the clinical relevance of *C. difficile* PCR-ribotype 176 and its capacity to spread within a healthcare facility.

## Findings

### Background

*Clostridium difficile* infection (CDI) is a major cause of antibiotic-associated diarrhoea and a significant burden to healthcare services worldwide [1]. Results of a pan-European epidemiological study in 2008 indicated that the Czech Republic has a relatively low CDI incidence (1.1 per 10,000 patient bed-days and 7.0 per 10,000 hospital admissions) [2], although a recent epidemiological study suggested a CDI incidence rate of 4.4 and 6.2 cases per 10,000 patient bed-days in 2011–12 and 2012–13, respectively [3].

In 2013, the high prevalence of PCR-ribotype 176 (n = 251; 40 %) was revealed by ribotyping of 624 *C. difficile* isolates from 11 Czech healthcare facilities [4] *C. difficile* ribotype 176 is thought to share many similarities to ribotype 027 [5–7] and it has been suggested that this type may be misdiagnosed as a ribotype 027 infection [8]. The long-term epidemic occurrence of *C. difficile* PCR-ribotype 176 was also reported in Poland [9, 10].

In response to the reported unfavourable global CDI epidemiological situation, including in Czech Republic, a 1-year study was initiated to monitor the incidence of CDI, clinical features and outcomes and to investigate the molecular characteristics of *C. difficile* isolates in patients with CDI hospitalised in the Internal Medicine department of University Hospital Motol, Prague, Czech Republic, from February 2013 to February 2014.

### Microbiological testing

Stool samples of 85 patients aged ≥18 years with three or more unformed stools per day were investigated at the Department of Medical Microbiology. CDI was laboratory diagnosed using the *C. difficile* Quik Chek Complete^®^ test (Alere) and *C. difficile* Alere and simultaneous toxin A/B positivity was detected in 24 samples (80 %). In six samples that were only GDH-positive but where patients had relevant clinical symptoms, the presence of toxigenic *C. difficile* was confirmed using PCR (GeneXpert^®^, Cepheid). Positive stool samples (GDH and toxin positive; GDH positive, toxin negative and PCR positive) were cultured anaerobically, after an alcohol shock treatment, on selective media (Oxoid); anaerobic culture was positive for *C. difficile* in 27/30 samples (90 %). Antibiotic susceptibility of *C. difficile* isolates to metronidazole and vancomycin was determined by E-test^®^ (BioMérieux) and minimum inhibitory concentrations for all *C. difficile* isolates ranged from 0.03–2 mg/L for metronidazole and 0.015–1 mg/L for vancomycin (Table 1). No isolates were found to be resistant to either metronidazole or vancomycin.

### *C. difficile* isolates molecular characterisation

PCR-ribotyping was performed according to the Standard Operating Protocol of ECDIS-net (http://www.ecdisnet.eu) using capillary electrophoresis after PCR amplification with primers previously described by Stubbs et al. [11]. Electrophoreograms were confirmed using the Webribo database [12]. PCR-ribotypes were identified for all 27 *C. difficile* isolates and 14 (51.9 %) belonged to ribotype 176. Other identified ribotypes were 012 (n = 2; 7.4 %), 014 (n = 2; 7.4 %), 001, 002, 005, 017, 020, 049, 078, 434 and 015 (all n = 1; 3.7 %).

The presence of toxin genes was determined by multiplex PCR with specific primers for *tcdA* (toxin A), *tcdB* (toxin B), *cdtA* and *cdtB* (binary toxin) [13]. All *C. difficile* isolates revealed presence of genes for production of toxins A/B, while genes for production of binary toxin (*cdtA*/*cdtB*), which has been associated with increased attachment to epithelial cells, increased virulence and higher recurrence rates [14–16] were only found in isolates of ribotypes 176 and 078 (15/27; 55.6 %). Summary of microbiological and molecular characteristics of C. difficile isolates is shown in Table 1.

The *tcdC* gene was amplified with primers C1 and C2 [17] and the obtained sequence was compared to NCBI reference sequence NC_009089.1. Two deletions (position 117, which introduces a frame-shift mutation leading to protein truncation [17], and 330–347) in the *tcdC* gene were found in all 14 ribotype 176 isolates. One isolate, ribotype 078, revealed 39-bp deletion from nucleotides 341–379 in the *tcdC* gene. No deletion in other 12 isolates was found. The precise function of the *tcdC* gene is not yet clear [18].

**Fig. 1:**
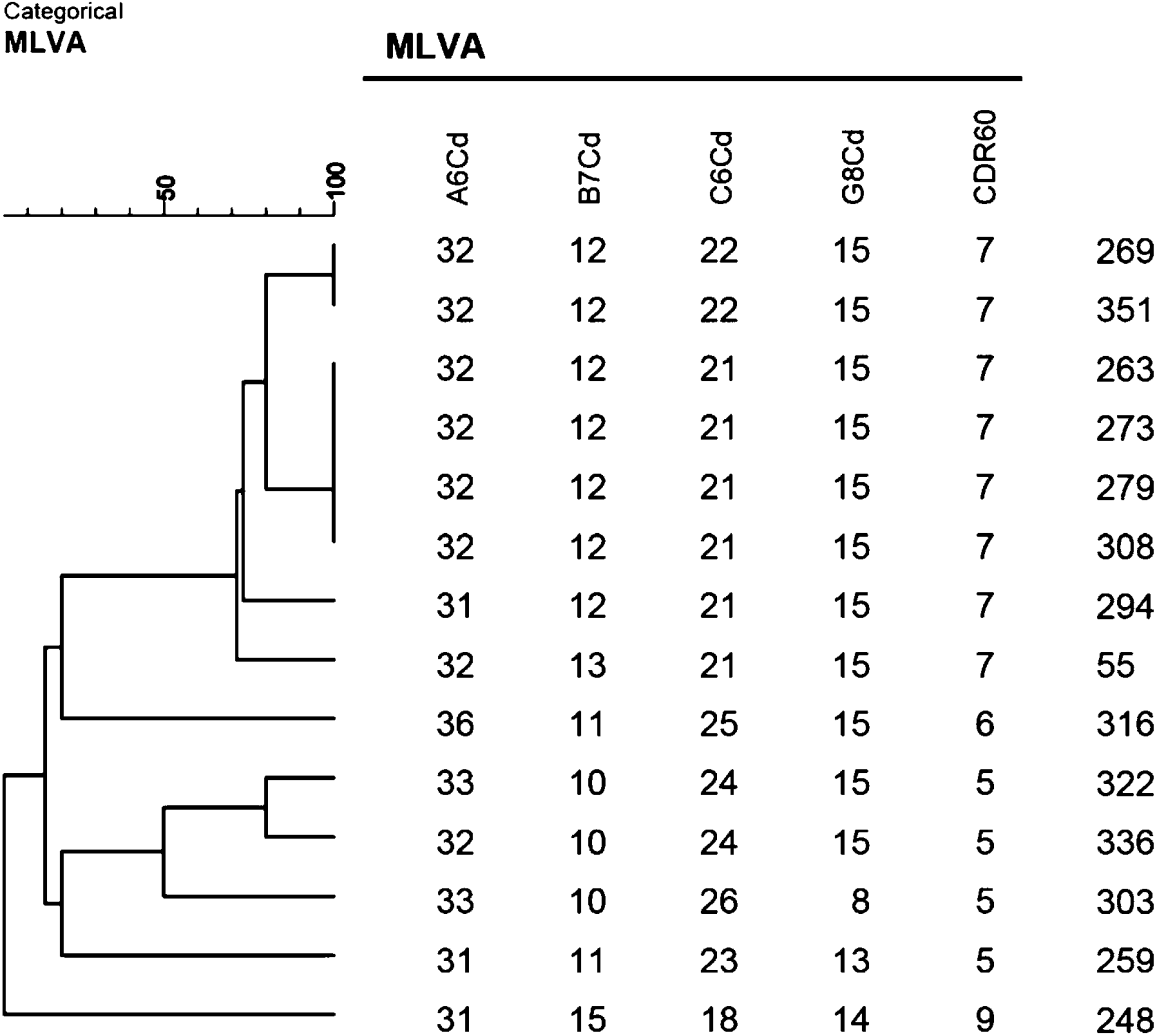
A Categorical MLVA of *C. difficile* ribotype 176 isolates (Bionumerics v5.0, Applied Maths)

**Fig. 2:**
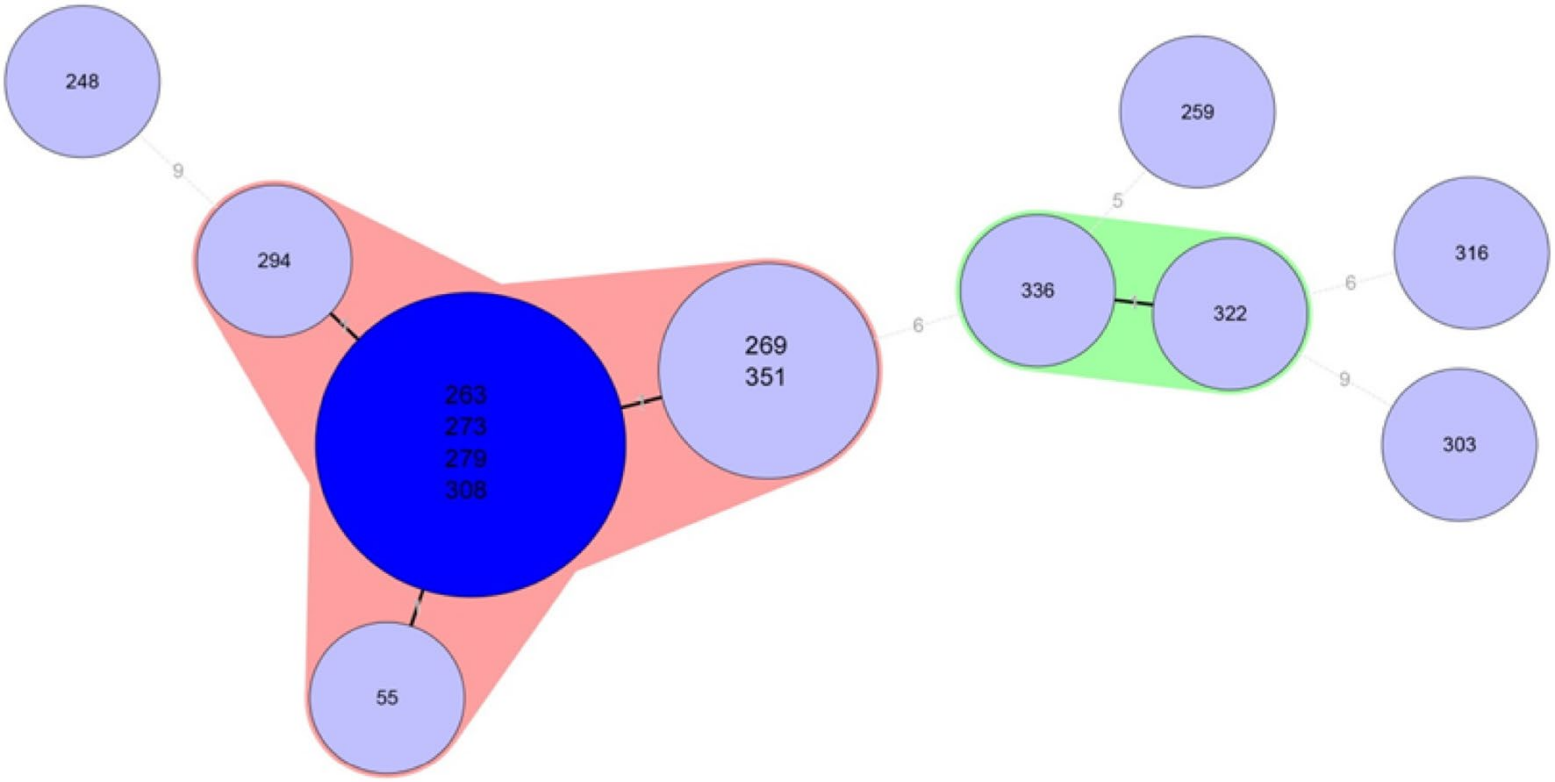
A Minimum Spanning Tree of *C. difficile* ribotype 176 isolates (Bionumerics v5.0, Applied Maths). The *numbers in the* *circles* represent *C. difficile* PCR-ribotype 176 isolate number. The *numbers in the* *lines* represent the sum of tandem repeat differences between isolates

Genetic relatedness among *C. difficile* ribotype 176 isolates was achieved using multi-locus-variable tandem repeats-analysis (MLVA). The number of tandem repeats were determined by Sanger sequencing in five previously published variable tandem repeat (VNTR) loci (A6Cd, B7Cd, C6Cd, G8Cd) [19, 20] and CDR60 [21]. A Minimum Spanning Tree (MST) was created by Bionumerics v5.0 (Applied Maths) using a Manhattan coefficient to calculate the summed tandem repeat difference (STRD). A cluster analysis using the categorical distance and unweighted pair group method with arithmetic mean algorithms was also applied. The number of tandem repeats for each locus is summarised in Fig. 1. MST identified two clonal complexes when STRD ≤1 (Fig. 2). The first clonal complex was formed from eight isolates (55, 269, 351, 263, 273, 279, 294 and 308). The second clonal complex consisted of two isolates (336 and 322). Between clonal complexes one and two, STDR = 6 were found. Isolate 248 revealed STDR = 9 to isolate 294 (CC1), isolate 259 showed STDR = 5 to isolate 336 (CC2), isolate 316 showed STDR = 6 and isolate 303 STDR = 9 to isolate 322 (CC2).

**Table 1:** Microbiological and molecular characteristics of *C. difficile* isolates

CDI case no.	Isolate no.	GDH	Toxin A/B	Anaerobic culture	PCR-ribotype	Toxin gene presence (A/B/bin)^a,b^	Vancomycin MIC (mg/L)	Metronidazole MIC (mg/L)
1	n/a	+	+	–	n/a	n/a	n/a	n/a
2	259	+	+	+	176	A/B/bin	0.25	0.25
3	263	+	+	+	176	A/B/bin	0.25	0.25
4	269	+	+	+	176	A/B/bin	0.5	0.5
5	205	+	+	–	n/a	n/a	n/a	n/a
6	273	+	+	+	176	A/B/bin	0.6	0.25
7	298	+	–	+	049	A/B	1	0.5
8	280	+	+	–	n/a	n/a	n/a	n/a
9	279	+	+	+	176	A/B/bin	1	0.25
10	294	+	+	+	176	A/B/bin	0.12	1
11	303	+	+	+	176	A/B/bin	0.25	0.5
12	277	+	–	+	001	A/B	0.5	0.5
13	304	+	+	+	014	A/B	0.25	0.5
14	44	+	–	+	002	A/B	0.25	0.5
15	307	+	+	+	017	A/B	0.25	0.25
16	308	+	+	+	176	A/B/bin	0.25	0.25
17	316	+	+	+	176	A/B/bin	0.5	0.25
18	320	+	–	+	012	A/B	0.5	0.5
19	319	+	+	+	012	A/B	0.5	0.12
20	322	+	+	+	176	A/B/bin	0.5	2
21	365	+	–	+	176	A/B/bin	0.5	2
22	323	+	+	+	020	A/B	0.5	0.5
23	325	+	+	+	015	A/B	0.5	0.25
24	331	+	+	+	078	A/B/bin	0.5	0.25
25	336	+	+	+	176	A/B/bin	0.5	1
26	351	+	+	+	176	A/B/bin	0.25	1
27	365	+	–	+	014	A/B	0.5	0.5
28	391	+	+	+	005	A/B	0.25	0.5
29	388	+	+	+	434	A/B	0.25	0.12
30	248	+	+	+	176	A/B/bin	0.12	0.5

*GDH* glutamate dehydrogenase, *MIC* minimum inhibitory concentration

^a^Toxin A/toxin B/binary toxin

^b^Primers used to amplify *tcdA* are located upstream of the repetitive region in the 3′-end. The TcdA-negative strains due to 3′-end deletion revealed positive PCR amplification [13]

The time intervals of hospitalisation of patients infected by *C. difficile* ribotype 176 did not overlap except for two patients. This finding suggests that the probable source of infection may have come from the hospital environment and, given the high incidence of this ribotype previously reported in the Czech Republic [4], it is possible that ribotype 176 is endemic in the country and this type has been introduced into the hospital environment on several occasions.

### Clinical and epidemiological data analysis

CDI was diagnosed in 30 patients (female n = 13, male n = 17; mean age 69.0 years). The overall CDI incidence in the Internal Medicine ward during the study period was calculated as 6.8 CDI cases per 10,000 patient bed-days and 50.6 CDI cases per 10,000 admissions which indicated a higher CDI incidence compared with recently reported rates [3].

Healthcare-associated CDI (HA-CDI) was diagnosed in 26 CDI cases (86.7 %) and community-associated CDI (CA-CDI) was diagnosed in four CDI cases (13.3 %). Severe CDI was diagnosed in 17 (56.7 %) patients according to the Horn’s index [22, 23] and 18 (60 %) according to the ATLAS score [24]. Antibiotic treatment prior to CDI diagnosis was noted for 83.3 % (25/30) of patients. The most commonly used antibiotics were aminopenicillins with beta-lactamase inhibitors (n = 12), fluoroquinolones (n = 12), broad-spectrum cephalosporins (n = 11), carbapenems (n = 4), piperacillin–tazobactam (n = 3) and aminoglycosides (n = 3). Administered CDI treatments, according to valid guidelines at the time of the study [25], were metronidazole (n = 10; 33.3 %), vancomycin (n = 3; 10.0 %), combined metronidazole and vancomycin (n = 13; 43.3 %), and metronidazole with other therapies (n = 1; 3.3 %). Three patients did not receive treatment for CDI. CDI recurrence within 3 months of treatment discontinuation was observed in 13.3 % (4/30) of patients and two received faecal transplant for recurrent disease. Mortality within 3 months after first CDI episode was 26.7 % (8/30); CDI was the cause of death in two cases 6.7 % (2/30) (Table 2).

**Table 2:** Study population and patient demographics (n = 30)

Patient characteristic	N (%)
Male	17 (56.7)
Age ≥ 65 years	22 (73.3)
HA-CDI	26 (86.7)
CA-CDI	4 (13.3)
Recurrent CDI	4 (13.3)
Severe CDI—Horn’s index	17 (56.7)
Severe CDI—Atlas score	18 (60)
Mortality within 3 months	8 (26.7)
CDI cause of death	2 (6.7)
Previous hospitalisation	13 (59.1)
Previous antibiotic use	25 (83.3)
Aminopenicillin/beta-lactamase inhibitors	12 (40)
Cephalosporines	11 (36.7)
Fluoroquinolones	12 (40)
Carbapenems	4 (13.3)
Piperacilin/tazobactam	3 (10)
Aminoglycosides	3 (10)

To assess the association between *C. difficile* ribotype and disease severity, the clinical outcomes of patients with ribotype 176 infections were compared to those with other ribotype infections (Table 3). Analysis of ATLAS scores and the Horn’s index found that 11/14 (78.6 %) patients with ribotype 176 infections had an ATLAS score of 6–9 or a Horn’s index score of 3 or 4 compared with 6/13 (46.2 %) and 7/13 (53.9 %) of patients with non-ribotype 176 infections. Furthermore, the mortality rate appeared to be higher in patients with ribotype 176 infections compared with non-ribotype 176 infections (35.7 versus 15.4 %). No significant ribotype-associated differences were noted in recurrence rates, ICU admission rates or prior antibiotic use (Table 3).

**Table 3:** Comparison of clinical outcomes in patients grouped by isolated *C. difficile* PCR-ribotype

Clinical outcome	Ribotype 176 (n = 14)		Other ribotypes (n = 13)	
	N	%	N	%
Horn’s index				
1			1	7.7
2	3	21.4	6	46.1
3	9	64.3	5	38.5
4	2	14.3	1	7.7
Atlas score				
1–2			2	15.4
3–5	3	21.4	4	30.8
6–7	9	64.3	5	38.5
8–9	2	14.3	2	15.4
Recurrent CDI within 3 months of first episode (Yes)	2	14.3	2	15.4
CDI in 8 weeks prior to admission (Yes)	1	7.1	1	7.7
Admitted to ICU (Yes)	3	21.4	3	23.1
Antibiotic treatment within 1 month prior to admission (Yes)	12	85.7	10	76.9
Mortality within 3 months of first CDI episode (Yes)	5	35.7	2	15.4

*Clostridium difficile* ribotype 027 strains are often thought to be associated with CDI outbreaks of increased disease severity [1, 5], but the clinical severity associated with ribotype 176 infections has not yet been studied in detail with exception of clinical data on ten patients, of whom 50 % had severe form of CDI, reported by Obuch-Woszczatyński et al. [9]. Our finding of a trend towards increased Horn’s index and ATLAS scores in patients with ribotype 176 infections compared with non-ribotype 176 infections provides some evidence to support the clinical importance of this ribotype. However, the small sample size of patients in this study indicates a need for further studies, incorporating a larger number of patients, to better understand the relative virulence of ribotype 176. The high incidence of epidemic *C. difficile* PCR-ribotype 176 in our study emphasises the importance of implementing continuous surveillance programmes for CDI at national and European level, including PCR ribotyping.
